# Impact of red blood cell transfusion volume on long-term mortality after transcatheter aortic valve replacement

**DOI:** 10.3389/fcvm.2025.1612385

**Published:** 2025-12-09

**Authors:** Yi Zhang, Shiqin Peng, Weiya Li, Yuan Feng, Yong Peng, Jiafu Wei, Zhengang Zhao, Yuanweixiang Ou, Mao Chen

**Affiliations:** 1 Department of Cardiology, West China Hospital, Sichuan University, Chengdu, China; 2 Laboratory of Cardiac Structure and Function, Institute of Cardiovascular Diseases, West China Hospital, Sichuan University, Chengdu, China; 3 Cardiac Structure and Function Research Key Laboratory of Sichuan Province, West China Hospital, Sichuan University, Chengdu, China

**Keywords:** transfusion, bleeding, mortality, transcatheter aortic valve replacement, transcatheter aortic valve implantation

## Abstract

**Backgrounds:**

The relationship between red blood cell (RBC) transfusion volume and long-term survival outcomes following transcatheter aortic valve replacement (TAVR) remains inadequately characterized. This study sought to investigate the clinical impact of perioperative transfusion and identify critical thresholds for transfusion volume in predicting mortality risk after TAVR.

**Methods:**

In this retrospective cohort analysis, patients undergoing TAVR at a tertiary cardiac center between April 2012 and September 2023 were consecutively enrolled and stratified by transfusion status. Multivariate Cox regression models were employed to identify prognostic factors for mortality. The primary outcome was all-cause mortality at 1-year post-TAVR.

**Results:**

Of 1,758 included patients, 141 (8.02%) required RBC transfusions. Transfused patients exhibited higher risk profiles, female predominance, advanced age, anemia, chronic kidney disease at baseline, and increased rates of life-threatening/major bleeding, stroke, and stage 3 acute kidney injury. These patients also demonstrated elevated 30-day and 1-year mortality rates. While transfusion status (*P* = 0.690) and anemia (*P* = 0.188) showed no independent association with 1-year mortality, total transfusion volume emerged as a significant independent predictor (adjusted hazard ratio 1.07, 95% CI: 1.02–1.12; *P* = 0.008), with 4.5 units identified as the optimal threshold for mortality risk stratification. Life-threatening/major bleeding events constituted the sole independent predictor of transfusion volumes exceeding 4.5 units (*P* = 0.039).

**Conclusions:**

Elevated transfusion volumes significantly correlate with increased long-term mortality risk in transfused TAVR recipients, primarily mediated by life-threatening hemorrhagic complications. These findings underscore the importance of implementing bleeding mitigation strategies to minimize transfusion requirements and improve clinical outcomes.

## 1 Introduction

Transcatheter aortic valve replacement (TAVR) has become established as a first-line treatment modality for symptomatic severe aortic stenosis, with continually broadening clinical applications that now include progressively younger patient cohorts. This paradigm shift in treatment eligibility underscores the growing imperative for meticulous perioperative management and enhanced risk assessment strategies tailored to contemporary practice patterns.

The hemoglobin (Hb) fluctuations observed during TAVR hospitalization predominantly stem from two interrelated factors: preexisting anemia and procedure-induced hemorrhagic events. Clinical data reveal that 11%–18% of elderly TAVR patients develop severe anemia—a condition strongly correlated with increased transfusion requirements, heightened incidence of acute kidney injury (AKI), and elevated long-term mortality risk (1). Furthermore, major bleeding complications (occurring in 5%–15% of cases) have been consistently associated with elevated mortality risks at both 30 days and 1 year post-procedure (2, 3). While red blood cell (RBC) transfusion serves as an essential therapeutic intervention in such scenarios, accumulating evidence paradoxically associates perioperative transfusions with increased short- and long-term mortality risks in TAVR recipients (4). This dichotomy highlights the urgent requirement for developing evidence-based transfusion algorithms that balance hematological stabilization with transfusion-related risks. Current knowledge gaps persist regarding the dose-response relationship between transfusion volumes and clinical outcomes, particularly in determining thresholds that optimize patient survival while minimizing complications.

Against this background, our study sought to investigate the prognostic impact of perioperative RBC transfusion volumes on 1-year survival post-TAVR, while concurrently establishing a clinically relevant threshold for transfusion volume to optimize risk stratification in transfused patients. This investigation provides novel insights into optimizing transfusion practices while advancing personalized outcome prediction in contemporary TAVR management.

## 2 Methods

### 2.1 Study population

This retrospective cohort study analyzed patients from a tertiary cardiac center serving a catchment area of over ten million residents in southwestern China. We consecutively enrolled individuals undergoing first-time TAVR between April 2012 and September 2023. All candidates underwent comprehensive preprocedural evaluations aligned with guideline recommendations, including clinical assessments, essential hematological profiling, echocardiography, and multidetector computed tomography (MDCT) (5). The heart team collaboratively determined TAVR eligibility through multidisciplinary case reviews. Exclusion criteria encompassed incomplete periprocedural hematological data, redo-TAVR cases, conversions to surgical intervention, or unsuccessful bioprosthesis implantation. Institutional ethics committee approval was obtained for this study.

### 2.2 TAVR procedure

Procedures followed a standardized protocol as previously detailed (6). Patients received general or local anesthesia, with heparin administration titrated to maintain activated clotting time (ACT) ≥250 s through intraprocedural monitoring. Vascular access strategies, predominantly femoral artery approaches, were optimized using preprocedural MDCT analysis. Valve selection included both self-expanding [Venus-A/Venus-A Plus (Venus MedTech), VitaFlow I/II (Microport), TaurusOne/TaurusElite (Peijia Medical), CoreValve/Evolut R/Evolut Pro (Medtronic), Prostyle (KingstronBio)] and balloon-expandable systems [Sapien XT/Sapien 3 (Edwards Lifesciences), Prizvalve (Newmed Medical)]. Predilation was selectively performed for severe valvular calcification, while postdilation addressed suboptimal valve expansion, paravalvular leakage, or elevated transaortic gradients. Procedural success was confirmed via echocardiography and aortic root angiography, with preclosure devices routinely deployed for vascular access management.

### 2.3 Hematological profiling

Serial hematological assessments were performed at standardized intervals during the TAVR hospitalization period, including complete blood counts, coagulation panels, and hepatic/renal/cardiac function biomarkers. Data were extracted from the institutional Hospital Information System, with baseline values reflecting initial post-admission measurements and discharge values representing final pre-discharge evaluations.

Transfusion decisions followed clinical guidelines, prioritizing Hb decline >3 g/dL post-TAVR or critical anemia (Hb <7 g/dL) (7). Transfusion volumes and administration timing were systematically recorded. Anemia was classified per World Health Organization criteria (Hb ≤13 g/dL for males, ≤12 g/dL for females) using baseline measurements (8). Participants were stratified by transfusion status for comparative analyses.

### 2.4 Clinical endpoints and event definitions

Baseline characteristics encompassed demographic parameters, comorbidities, and laboratory profiles, supplemented by Society of Thoracic Surgeons Predicted Risk of Mortality (STS-PROM) scores and New York Heart Association (NYHA) functional classifications. Early postoperative complications associated with 1-year mortality were adjudicated using Valve Academic Research Consortium-3 criteria, including (9–11):

(i) Moderate-severe aortic regurgitation (central or paravalvular)
(ii) Stroke confirmed by neurological deficits with neuroimaging correlation
(iii) Life-threatening/major bleeding (Hb drop >3 g/dL requiring ≥2 RBC units)
(iv) Stage 3 AKI (serum creatinine >300% baseline or ≥354 µmol/L with acute ≥44 µmol/L rise)

The primary endpoint was 1-year all-cause mortality, with 30-day mortality as a secondary endpoint.

### 2.5 Statistical analysis

Continuous variables were expressed as mean ± standard deviation (SD) or median [interquartile range (IQR)], categorical variables as counts (%). Intergroup comparisons utilized Student's *t*-tests, Mann–Whitney *U* tests, *χ*^2^ tests, or Fisher's exact tests as appropriate. Mortality predictors were identified through multivariable Cox regression (hazard ratios [HRs] with 95% confidence intervals ([CIs]). Transfusion volume determinants were analyzed via logistic regression [odds ratios (ORs) with 95% CIs]. Variables with *p* < 0.05 in univariate analyses entered multivariable models. Transfusion volume thresholds were optimized using maximally selected rank statistics.

Sample size adequacy for regression modeling was verified via the events-per-variable (EPV) principle:

$$N=\frac{EPV\times P}{d}$$

where *N* = required sample size, *EPV* = 10 events per predictor, *P* = predictor count, and *d* = observed mortality rate. Analyses were conducted in R v4.4.0 and Zstats 1.0 (https://www.zstats.net).

## 3 Results

### 3.1 Cohort characteristics and transfusion profile

The study cohort comprised 1,758 patients (median age 73.39 years; 41.7% female) undergoing TAVR, with baseline demographics and procedural outcomes detailed in Table 1. The median baseline Hb was 12.80 g/dL, and anemia prevalence was 42.05% (738/1758).

**Table 1:** Baseline characteristics and clinical outcomes of all patients who underwent transcatheter aortic valve replacement divided by RBC transfusion during hospitalization.^a^

Variables	Total (*n* = 1,758)	No RBC transfusion (*n* = 1,617)	RBC transfusion (*n* = 141)	*P* ^b^
Baseline characteristics				
Female sex, *n* (%)	733 (41.70)	654 (40.45)	79 (56.03)	**<0**.**001**
BMI//kg·m^−2^, M (Q_1_, Q_3_)	22.67 (20.40, 24.98)	22.81 (20.45, 25.05)	21.62 (19.04, 23.81)	**<0**.**001**
Age/year, M (Q_1_, Q_3_)	73.39 (68.05, 78.19)	73.17 (67.84, 77.88)	76.22 (71.50, 80.70)	**<0**.**001**
STS-PROM/%, M (Q_1_, Q_3_)	3.29 (2.07, 6.10)	3.13 (1.99, 5.70)	6.38 (3.45, 9.26)	**<0**.**001**
Baseline Hb/g·dL^−1^, M (Q_1_, Q_3_)	12.80 (11.50, 13.90)	12.90 (11.80, 14.00)	9.86 (7.80, 11.80)	**<0**.**001**
Aortic V_max_/m·s^−1^, M (Q_1_, Q_3_)	4.60 (4.10, 5.20)	4.60 (4.10, 5.20)	4.80 (4.20, 5.40)	0.092
LVEF/%, M (Q_1_, Q_3_)	60.00 (43.50, 67.00)	60.00 (44.00, 67.00)	60.50 (43.00, 67.00)	0.872
CAD, *n* (%)	617 (35.10)	569 (35.19)	48 (34.04)	0.784
DM, *n* (%)	311 (17.69)	291 (18.00)	20 (14.18)	0.255
Anemia, *n* (%)	738 (42.05)	627 (38.82)	111 (79.29)	**<0**.**001**
CKD, *n* (%)	507 (28.84)	445 (27.52)	62 (43.97)	**<0**.**001**
NYHA III–IV, *n* (%)	1,390 (80.30)	1,263 (79.18)	127 (93.38)	**<0**.**001**
HTN, *n* (%)	740 (42.09)	686 (42.42)	54 (38.30)	0.341
COPD, *n* (%)	211 (12.10)	191 (11.86)	20 (15.04)	0.280
AF, *n* (%)	259 (14.73)	235 (14.53)	24 (17.02)	0.424
Cancer, *n* (%)	81 (4.64)	79 (4.90)	2 (1.50)	0.073
Post-procedural information				
Aortic *V*_max_/m·s^−1^, M (Q_1_, Q_3_)	2.20 (1.90, 2.60)	2.20 (1.90, 2.60)	2.20 (1.90, 2.60)	0.719
Discharged Hb/g·dL^−1^, M (Q_1_, Q_3_)	11.20 (9.80, 12.60)	11.40 (10.10, 12.70)	8.80 (7.80, 9.73)	**<0**.**001**
Discharged albumin/g·L^−1^, M (Q_1_, Q_3_)	37.40 (34.80, 39.80)	37.60 (35.00, 39.92)	34.50 (30.80, 37.50)	**<0**.**001**
Post-TAVR AR ≥ moderate, *n* (%)	117 (6.67)	107 (6.63)	10 (7.19)	0.796
Stroke, *n* (%)	48 (2.86)	39 (2.51)	9 (7.38)	**0**.**005**
Life-threatening/major bleeding, *n* (%)	280 (16.26)	188 (11.89)	92 (65.25)	**<0**.**001**
Stage 3 AKI, *n* (%)	22 (1.25)	17 (1.05)	5 (3.55)	**0**.**031**
Hospitalization duration after TAVR/day, M (Q_1_, Q_3_)	7.00 (6.00, 9.00)	7.00 (6.00, 9.00)	9.00 (7.00, 13.00)	**<0**.**001**
Transfusion volume/unit, M (Q_1_, Q_3_)	3.50 (2.00, 4.50)	/	3.50 (2.00, 4.50)	/
Follow-up information				
30-day all-cause mortality, *n* (%)	54 (3.07)	29 (1.79)	25 (17.73)	**<0**.**001**
1-year all-cause mortality, *n* (%)	90 (5.12)	60 (3.71)	30 (21.28)	**<0**.**001**

a M, median; Q_1_, 1st quartile; Q_3_, 3rd quartile; RBC transfusion, red blood cell transfusion; BMI, body mass index; STS-PROM, society of thoracic surgeons predicted risk of mortality; Hb, hemoglobin; Aortic V_max_, maximun velocity across aortic valve; LVEF, left ventricular ejection fraction; CAD, coronary artery disease; DM, diabetes mellitus; CKD, chronic kidney disease; NYHA, New York heart association; HTN, hypertension; COPD, chronic obstructive pulmonary disease; AF, atrial fibrillation; TAVR, transcatheter aortic valve replacement; AR, central and paravalvular aortic regurgitation; AKI, acute kidney injury.

b Bold values were statistically significant at *P* < 0.05.

Periprocedural RBC transfusions were administered to 8.02% (141/1758) of patients. Transfused recipients received a median of 3.50 units (IQR: 2.00–4.62), accompanied by a median Hb decline from 9.86 g/dL at baseline to 8.80 g/dL pre-discharge. Transfused patients exhibited significantly elevated baseline risk profiles compared to non-transfused counterparts, including older age (76.22 vs. 73.17 years; *P* < 0.001), higher STS-PROM scores (6.38% vs. 3.13%; *P* < 0.001), and greater prevalence of female sex (56.03% vs. 40.45%; *P* < 0.001), chronic kidney disease (CKD) (43.97% vs. 27.52%; *P* < 0.001), NYHA III–IV classification (93.38% vs. 79.18%; *P* < 0.001), and baseline anemia (79.29% vs. 38.82%; *P* < 0.001). Postprocedural complications were disproportionately observed in transfused patients, including stroke (7.38% vs. 2.51%; *P* = 0.005), life-threatening/major bleeding (65.25% vs. 11.89%; *P* < 0.001), and stage 3 acute kidney injury (3.55% vs. 1.05%; *P* = 0.031). Mortality rates were markedly elevated in the transfused cohort at both 30 days (17.73% vs. 1.79%; *P* < 0.001) and 1 year (21.28% vs. 3.71%; *P* < 0.001).

### 3.2 Mortality determinants in the overall cohort

Univariate Cox regression identified multiple factors associated with 1-year mortality (Table 2): advancing age (HR = 1.06, 95% CI: 1.03–1.10; *P* < 0.001), elevated STS-PROM (HR = 1.10, 95% CI: 1.08–1.13; *P* < 0.001), reduced left ventricular ejection fraction (LVEF) (HR = 0.98, 95% CI: 0.97–0.99; *P* = 0.005), anemia (HR = 1.72, 95% CI: 1.13–2.61; *P* = 0.011), CKD (HR = 2.16, 95% CI: 1.42–3.26; *P* < 0.001), stroke (HR = 3.45, 95% CI: 1.38–8.63; *P* = 0.008), life-threatening/major bleeding (HR = 3.66, 95% CI: 2.39–5.61; *P* < 0.001), stage 3 AKI (HR = 12.44, 95% CI: 5.99–25.85; *P* < 0.001), transfusion (HR = 6.50, 95% CI: 4.19–10.07; *P* < 0.001), and lower discharge albumin (HR = 0.83, 95% CI: 0.80–0.86; *P* < 0.001).

**Table 2:** Cox-regression of 1-year mortality of all patients who underwent transcatheter aortic valve replacement.^a^

Variables	Univariate Cox regression		Multivariate Cox regression^b^	
	HR (95%CI)	*P*	HR (95%CI)	*P*
Female sex	0.80 (0.52–1.23)	0.313	/	/
BMI	0.94 (0.89–1.01)	0.079	/	/
Age	1.06 (1.03–1.10)	**<**.**001**	1.03 (0.99–1.08)	0.113
STS-PROM	1.10 (1.08–1.13)	**<**.**001**	1.06 (1.02–1.11)	**0**.**003**
LVEF	0.98 (0.97–0.99)	**0**.**005**	1.00 (0.98–1.02)	0.818
CAD	0.98 (0.63–1.51)	0.913		
DM	0.85 (0.48–1.50)	0.569		
Anemia	1.72 (1.13–2.61)	**0**.**011**	1.54 (0.81–2.92)	0.188
CKD	2.16 (1.42–3.26)	**<**.**001**	1.01 (0.54–1.87)	0.985
NYHA III–IV	1.74 (0.93–3.27)	0.085	/	/
HTN	1.14 (0.75–1.73)	0.536	/	/
COPD	1.32 (0.71–2.45)	0.373	/	/
AF	1.28 (0.74–2.19)	0.375	/	/
Cancer	1.53 (0.62–3.78)	0.359	/	/
Post-TAVR AR ≥ moderate	1.05 (0.46–2.40)	0.910	/	/
Stroke	3.45 (1.38–8.63)	**0**.**008**	3.30 (1.29–8.43)	**0**.**013**
Life-threatening/major bleeding	3.66 (2.39–5.61)	**<**.**001**	1.05 (0.51–2.18)	0.892
Stage 3 AKI	12.44 (5.99–25.85)	**<**.**001**	9.19 (3.63–23.27)	**<**.**001**
Transfusion	6.50 (4.19–10.07)	**<**.**001**	1.18 (0.52–2.66)	0.690
Discharged albumin	0.83 (0.80–0.86)	**<**.**001**	0.88 (0.83–0.94)	**<**.**001**

a HR, hazard ratio; CI, confidence interval; BMI, body mass index; STS-PROM, society of thoracic surgeons predicted risk of mortality; LVEF, left ventricular ejection fraction; CAD, coronary artery disease; DM, diabetes mellitus; CKD, chronic kidney disease; NYHA, New York heart association; HTN, hypertension; COPD, chronic obstructive pulmonary disease; AF, atrial fibrillation; TAVR, transcatheter aortic valve replacement; AR, central and paravalvular aortic regurgitation; AKI, acute kidney injury.

b Estimated sample = (10*10)/0.0512 = 1,953. Bold values were statistically significant at *P* < 0.05.

Multivariable analysis (sample size adequacy: *N* = 1,953) demonstrated independent mortality associations for STS-PROM (HR = 1.06, 95% CI: 1.02–1.11; *P* = 0.003), stroke (HR = 3.30, 95% CI: 1.29–8.43; *P* = 0.013), stage 3 AKI (HR = 9.19, 95% CI: 3.63–23.27; *P* < 0.001), and discharge albumin (HR = 0.88, 95% CI: 0.83–0.94; *P* < 0.001). Transfusion status (HR: 1.18, 95% CI: 0.52–2.66, *P* = 0.690), life-threatening/major bleeding events (HR: 1.05, 95% CI: 0.51–2.18, *P* = 0.892), anemia (HR: 1.54, 95% CI: 0.81–2.92, *P* = 0.188), CKD (HR: 1.01, 95% CI: 0.54–1.87, *P* = 0.985), LVEF (HR: 1.00, 95% CI: 0.98–1.02, *P* = 0.818), and age (HR: 1.03, 95% CI: 0.99–1.08, *P* = 0.113) lost statistical significance in adjusted models.

### 3.3 Mortality determinants in transfused patients

Among transfused recipients (Table 3), univariate analysis associated 1-year mortality with STS-PROM (HR = 1.06, 95% CI: 1.02–1.09; *P* < 0.001), LVEF (HR = 0.98, 95% CI: 0.95–0.99; *P* = 0.045), discharge albumin (HR = 0.86, 95% CI: 0.82–0.91; *P* < 0.001), and transfusion volume (HR = 1.07, 95% CI: 1.03–1.10; *P* < 0.001). Multivariable modeling (sample size adequacy: *N* = 188) confirmed independent prognostic value for STS-PROM (HR = 1.06, 95% CI: 1.02–1.09; *P* = 0.002), discharge albumin (HR = 0.88, 95% CI: 0.83–0.93; *P* < 0.001), and transfusion volume (HR = 1.07, 95% CI: 1.02–1.12; *P* = 0.008). LVEF (HR: 1.00, 95% CI: 0.97–1.03, *P* = 0.799) did not retain significance in adjusted analysis.

**Table 3:** Cox-regression of 1-year mortality of patients who received transfusion during transcatheter aortic valve replacement hospitalizaiton.^a^

Variables	Univariate Cox regression		Multivariate Cox regression^b^	
	HR (95%CI)	*P*	HR (95%CI)	HR (95%CI)
Female sex	0.79 (0.38–1.61)	0.510	/	/
BMI	1.03 (0.92–1.14)	0.639	/	/
Age	1.03 (0.98–1.09)	0.202	/	/
STS-PROM	1.06 (1.02–1.09)	**<**.**001**	1.06 (1.02–1.09)	**0**.**002**
LVEF	0.98 (0.95–0.99)	**0**.**045**	1.00 (0.97–1.03)	0.799
CAD	0.98 (0.46–2.09)	0.956	/	/
DM	1.15 (0.44–3.01)	0.774	/	/
Anemia	0.53 (0.24–1.15)	0.106	/	/
CKD	1.74 (0.84–3.58)	0.133	/	/
NYHA III–IV	1.00 (0.24–4.21)	0.997	/	/
HTN	1.26 (0.61–2.60)	0.529	/	/
COPD	1.49 (0.55–4.03)	0.427	/	/
AF	0.32 (0.08–1.33)	0.115	/	/
Cancer	0.00 (0.00 ∼ Inf)	0.997	/	/
Post-TAVR AR ≥ moderate	0.49 (0.07–3.62)	0.486	/	/
Stroke	1.17 (0.15–9.08)	0.879	/	/
Life-threatening/major bleeding	1.51 (0.67–3.38)	0.321	/	/
Stage 3 AKI	2.56 (0.61–10.78)	0.200	/	/
Discharged albumin	0.86 (0.82–0.91)	**<**.**001**	0.88 (0.83–0.93)	**<**.**001**
Multiple times of transfusion	1.44 (0.68–3.09)	0.342	/	/
Transfusion volume	1.07 (1.03–1.10)	**<**.**001**	1.07 (1.02–1.12)	**0**.**008**
Discharged Hb	0.98 (0.96–1.01)	0.203	/	/

a HR, hazard ratio; CI, confidence interval; BMI, body mass index; STS-PROM, society of thoracic surgeons predicted risk of mortality; LVEF, left ventricular ejection fraction; CAD, coronary artery disease; DM, diabetes mellitus; CKD, chronic kidney disease; NYHA, New York heart association; HTN, hypertension; COPD, chronic obstructive pulmonary disease; AF, atrial fibrillation; TAVR, transcatheter aortic valve replacement; AR, central and paravalvular aortic regurgitation; AKI, acute kidney injury; Hb, hemoglobin.

b Estimated sample = (10*4)/0.2128 = 188. Bold values were statistically significant at *P* < 0.05.

### 3.4 Transfusion volume threshold for mortality risk

**Figure 1:**
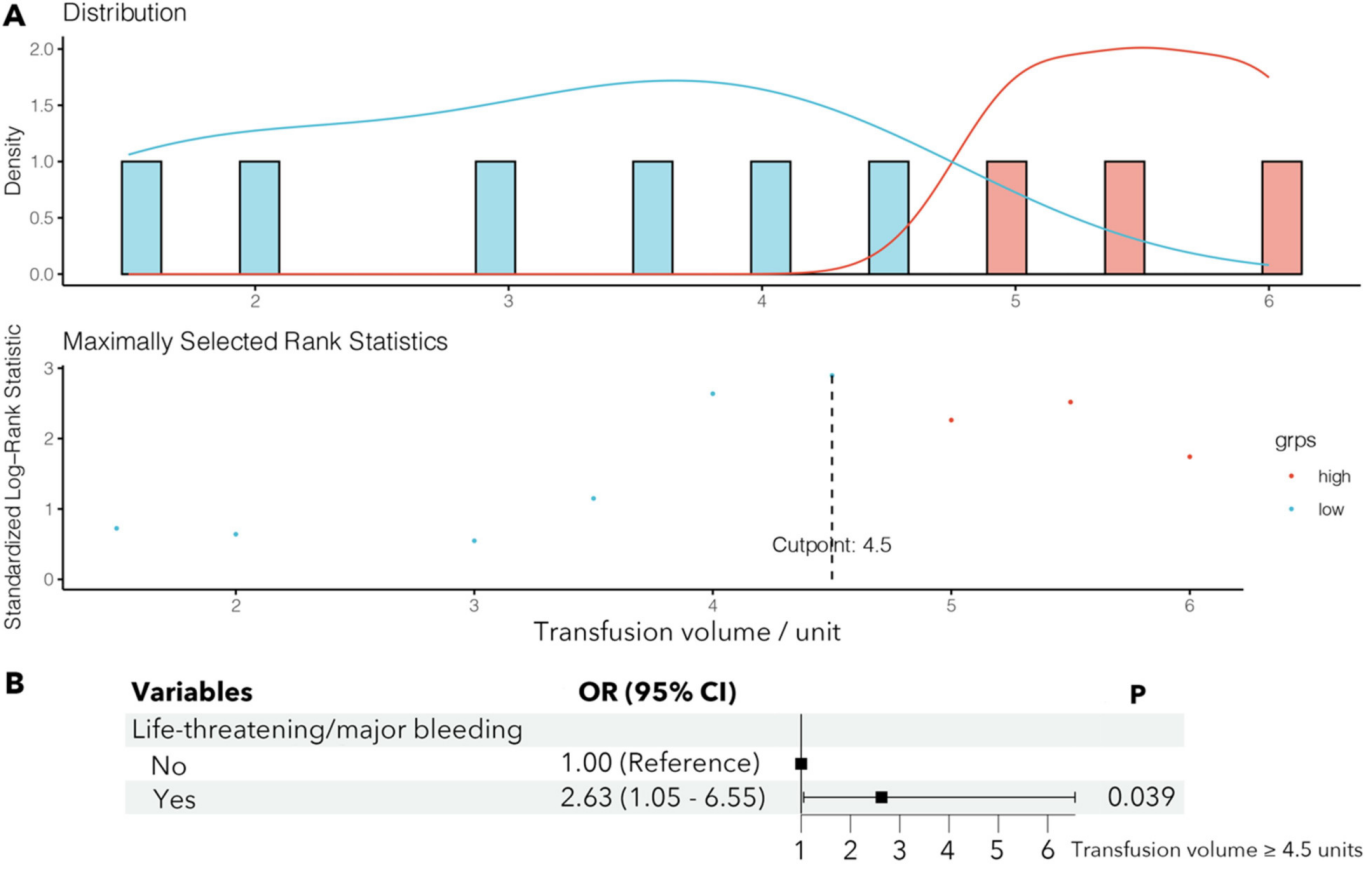
Maximally selected rank statistics of transfusion volume optimized for 1-year survival discrimination among transfused patients **(A)** and the logistic regression of predictors of high transfusion volume **(B)**. grps, groups; OR, odds ratio; CI, confidence interval.

**Figure 2:**
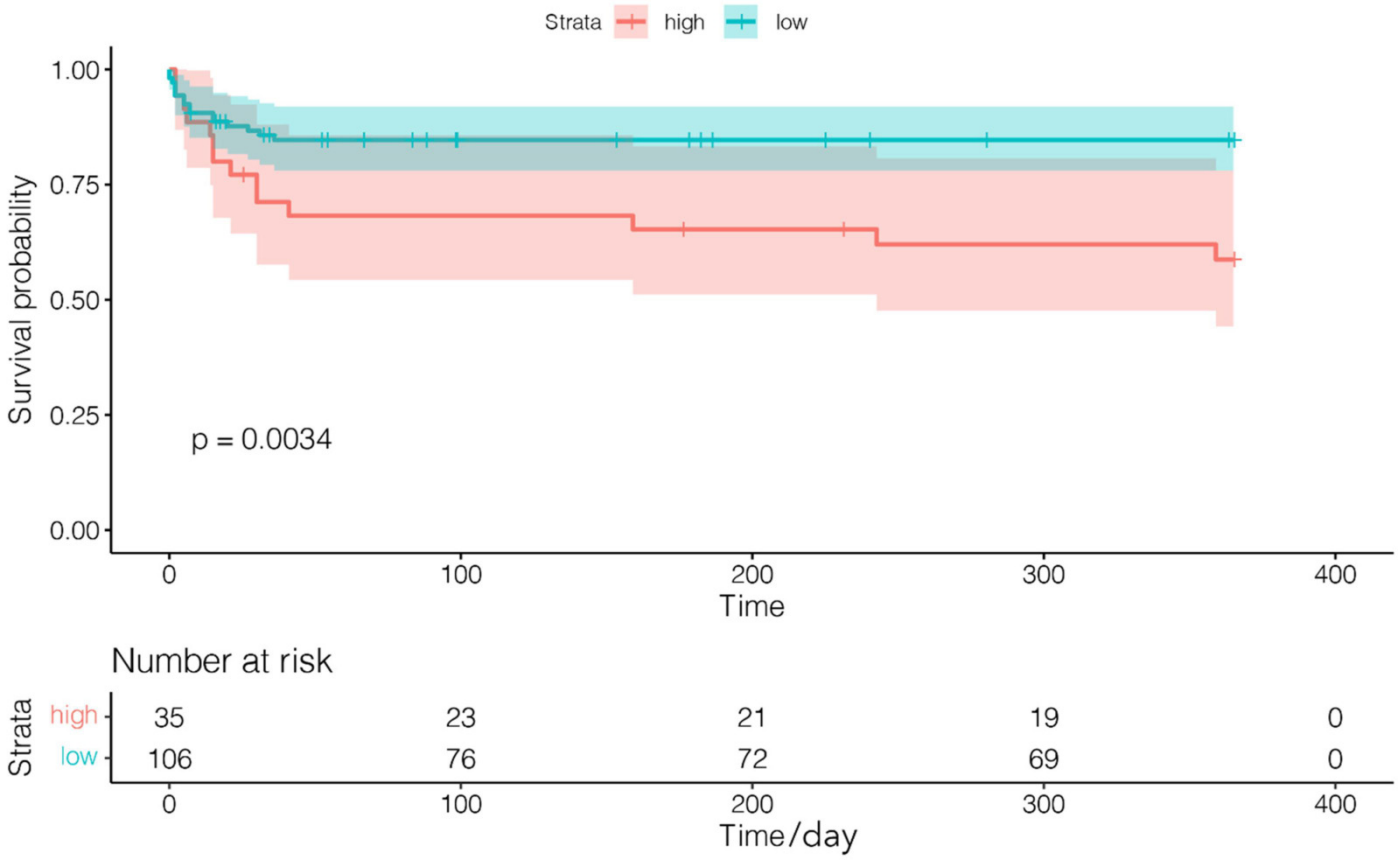
Survival curves of patients who received transfusion volume <4.5 units (low) and ≥4.5 units (high) during transcatheter aortic valve replacement hospitalization.

Maximally selected rank statistics identified 4.5 RBC units as the optimal threshold for mortality stratification in transfused patients (Figure 1A). Life-threatening/major bleeding independently predicted transfusion volumes exceeding this cutoff (OR = 2.63, 95% CI: 1.05–6.55; *P* = 0.039) (Figure 1B). Survival curves demonstrated significantly lower 1-year mortality in transfused patients receiving <4.5 units vs. ≥4.5 units (log-rank *P* = 0.0034; Figure 2).

## 4 Discussion

To our knowledge, this investigation represents the first comprehensive evaluation of transfusion volume thresholds for mortality risk stratification in transcatheter aortic valve replacement (TAVR) recipients. Our principal findings reveal three critical insights: (i) Transfused patients demonstrated distinct clinical characteristics including advanced age, female predominance, elevated baseline comorbidities (anemia, renal impairment, higher STS-PROM scores), and heightened susceptibility to perioperative complications such as cerebrovascular events, major hemorrhage, and stage 3 acute kidney injury, culminating in significantly increased 30-day and 1-year mortality rates; (ii) While transfusion status itself showed no independent prognostic significance in the overall TAVR cohort, cumulative transfusion volumes emerged as a robust independent predictor of 1-year mortality among transfused patients; (iii) Life-threatening/major bleeding episodes served as the exclusive determinant of high-volume transfusions (>4.5 units).

Reported transfusion rates in contemporary TAVR cohorts range from 6.7% to 17.3% (12–14), aligning closely with our observed rate of 8.02%. Despite significant procedural advancements in recent years, transfusion requirements persist in approximately 7% of cases (12). Our analysis corroborates previous reports identifying multifactorial transfusion predictors including both procedural factors (bleeding complications, pericardial effusion, emergency circulatory support) and patient-specific characteristics (pre-existing anemia, advanced age, female sex, renal dysfunction) (12, 13, 15). The heightened vascular complication risk in female patients, potentially related to smaller vessel anatomy and differential anticoagulation responses, may partially explain the gender disparity in transfusion needs. Contemporary evidence consistently associates perioperative transfusions with increased short- and long-term mortality, as well as post-TAVR complications including acute kidney injury and stroke (13–18). Our findings reinforce these observations through demonstrated correlations between transfusion events and elevated rates of mortality, neurological events, hemorrhagic complications, and renal impairment. These results emphasize the critical need for implementing multimodal blood conservation protocols to optimize TAVR outcomes.

The primary clinical indications for RBC transfusion in TAVR patients center on anemia management and bleeding control, suggesting two strategic avenues for preventive intervention. Notably, longitudinal analysis has revealed spontaneous hemoglobin recovery in 40% of anemic patients at 1-year follow-up—a phenomenon independent of transfusion practices and associated with improved survival outcomes. This hematological recovery pattern mirrors pathophysiological mechanisms observed in Heyde's syndrome, where high-gradient aortic stenosis induces von Willebrand factor abnormalities and subsequent gastrointestinal angiodysplasia-related anemia (19). The post-TAVR hemoglobin normalization observed in our cohort suggests that valvular hemodynamic correction may reverse anemia. Furthermore, preprocedural optimization strategies including iron repletion, erythropoiesis-stimulating agents, and nutritional support could reduce transfusion dependency while improving clinical outcomes (20). To address bleeding complications, comprehensive preventive measures incorporating ultrasound-guided vascular access, crossover balloon occlusion techniques, weight-adjusted anticoagulation regimens, and real-time hemostasis monitoring systems have been proposed (21).

The development of standardized transfusion thresholds remains particularly challenging in TAVR populations characterized by advanced age and multimorbidity (22). Current guidelines recommend restrictive transfusion strategies (Hb, 7–8 g/dL) for hemodynamically stable patients (23), with each RBC unit (350 mL) typically raising hemoglobin by 1 g/dL in an adult with stable blood volume. Our results found an independently higher risk of transfusion volume ≥4.5 units on 1-year mortality after TAVR, indicating a maximal increasement of Hb of 4.5 g/dL if receiving RBC transfusion of relatively risk controllable. Given the median discharge hemoglobin level of 8.8 g/dL in our cohort, these findings advocate for rigorous adherence to conservative transfusion protocols to mitigate mortality risks.

This study has several methodological limitations. First, while the single-center design introduces potential selection bias, the inclusion of a large, geographically diverse population from a tertiary referral center enhances generalizability. Second, limited sample size restricted comprehensive multivariate analysis of 30-day mortality endpoints, necessitating validation through multicenter collaborations. Third, the observational nature of our data precludes definitive causal interpretations between transfusion practices and clinical outcomes, and missing data, though generally minimal, may also introduce bias. Fourth, other clinical outcomes, such as functional status, were not evaluated due to a lack of systematic data collection. Future studies would benefit from incorporating functional assessments to provide a more comprehensive understanding of post-TAVR recovery.

## 5 Conclusion

Our analysis demonstrates that transfusion requirement and baseline anemia status do not independently predict 1-year mortality post-TAVR. However, high transfusion volumes (optimal cutoff: 4.5 units) emerged as a significant independent mortality predictor in the transfused cohort, with life-threatening/major bleeding serving as the sole determinant of such transfusion requirements. These findings underscore the imperative for implementing comprehensive bleeding prevention strategies and adopting restrictive transfusion protocols to optimize clinical outcomes in TAVR patients.

## Data Availability

The raw data supporting the conclusions of this article will be made available by the authors, without undue reservation.
